# First Vibrational Fingerprint of *Parietaria judaica* Protein via Surface-Enhanced Raman Spectroscopy

**DOI:** 10.3390/bios15030182

**Published:** 2025-03-13

**Authors:** Dario Morganti, Valeria Longo, Antonio Alessio Leonardi, Alessia Irrera, Paolo Colombo, Barbara Fazio

**Affiliations:** 1 CNR IMM-ME, Institute for Microelectronics and Microsystems, Viale F.S. d’Alcontres 31, I-98166 Messina, Italy; dario.morganti@cnr.it (D.M.); alessia.irrera@cnr.it (A.I.); 2 CNR DSFTM, Department of Physical Sciences and Technologies of Matter, Piazzale Aldo Moro, 7, I-00185 Roma, Italy; 3 CNR IRIB-PA, Institute for Biomedical Research and Innovation, Via U. La Malfa 153, I-90146 Palermo, Italy; valeria.longo@cnr.it; 4 Department of Chemical, Biological, Pharmaceutical, and Environmental Sciences (ChiBioFarAm), University of Messina, Viale F.S. d’Alcontres 31, I-98166 Messina, Italy; antonioalessio.leonardi@unime.it

**Keywords:** surface-enhanced Raman spectroscopy, silver dendrites, allergenic protein, *Parietaria judaica*

## Abstract

Accurate identification and characterization of allergenic proteins at the molecular level are essential for pinpointing the specific protein structures responsible for allergic reactions, thus advancing the development of precise diagnostic tests. Significant efforts have been focused on novel experimental techniques aimed at deepening the understanding of the underlying molecular mechanisms of these reactions. In this work, we show, for the first time to our knowledge, the unique Raman fingerprint of three *Parietaria judaica* (Par j) allergenic proteins. These proteins are typically present in pollen and are known to trigger severe respiratory diseases. In our research, we further exploited the surface-enhanced Raman scattering (SERS) effect from an Ag dendrite substrate. This approach provided better discrimination and a comprehensive analysis of the proteins Par j 1, 2, and 4 in hydration conditions, enabling rapid differentiation between them through a spectroscopic study.

## 1. Introduction

An allergic reaction is an exaggerated immune response to a substance, known as an allergen, that is typically harmless to most people [1]. When a subject with a sensitized immune system encounters an allergen, their body recognizes it as a threat and, by binding to immunoglobulin E antibodies, triggers an immediate immune response defined as hypersensitive type I reaction [2]. Therefore, the identification of allergenic proteins plays a pivotal role in public health, since it fosters research into allergy mechanisms, treatment options, and preventive strategies [3]. The *Parietaria* spp. is one of the most relevant sources of allergenic proteins in the Mediterranean area, causing severe respiratory symptoms such as rhinitis, conjunctivitis, and even asthma, which can range from mild discomfort to severe, life-threatening anaphylaxis [4]. Its allergenic composition has been investigated in detail and all the allergenic components have been identified by means of DNA recombinant technology [5]. Immunological characterization has shown that the families of the *Parietaria judaica* 1 and 2 (Par j 1, Par j 2) allergens are two major allergenic components of this pollen belonging to the family of nonspecific lipid transfer proteins [6]. On the other hand, the Par j 3 and Par j 4 allergens represent two minor cross-reactive allergenic molecules belonging to the family of the profilin and the calcium-binding protein, respectively [7].

Scientific and technological advances have shifted the focus toward in vitro allergy testing. Expanding knowledge of allergens at the molecular level has enabled the characterization of sensitization profiles and the identification of specific molecules triggering immunoglobulin E (IgE) production, using a technology called component-resolved diagnostics (CRD). While traditional in vitro allergen diagnostics rely on extracts, this approach often struggles to differentiate cross-reactivity and identify major allergen components. The accurate identification of allergenic proteins and their potential cross-reactivities is essential for optimal allergy management, though it remains challenging outside specialist centers. In the last two decades, it has been clearly demonstrated that the use of allergenic recombinant derivatives can substitute naturally derived allergens, and, for these reasons, they represent a tool of paramount importance for allergic patients to manage their allergies. In this view, the use of purified allergens has radically changed allergy diagnostics particularly for the study of allergen cross-reactivities. Clinical diagnostic tools rely on very well-characterized in vivo methods like the “skin prick test”, ex vivo techniques such as the “basotest”, and in vitro assays like “ELISA” (enzyme-linked immunosorbent assay) [8], all conducted in clinical settings and by skilled personnel. There are several types of singleplex and multiplex platforms using recombinant or purified natural molecular allergens, each with advantages and disadvantages. However, the development of novel detection assays that may be used outside specialized facilities are still a challenging topic in this field and for application in environmental and food detection technologies. For instance, allergen detection is crucial for ensuring food safety and protecting individuals with food allergies. Therefore, continuous effort is dedicated to developing new techniques or integrating existing strategies for the detection of allergenic molecules.

Label-free techniques, such as Raman spectroscopy, generally provide valuable insights into protein structure and properties, including secondary and tertiary structures, and can detect conformational changes induced by chemical environments or interactions with other proteins or DNA [9,10], thereby revealing underlying functional mechanisms. However, despite its utility in discerning molecules, including complex biomolecules, and studying their activity, Raman spectroscopy often faces the challenge of weak signals, making the detection of small quantities of molecular samples more difficult. In such cases, surface-enhanced Raman spectroscopy (SERS) emerges as an intriguing alternative application of the Raman technique [11,12,13]. SERS amplifies spectral intensities by leveraging the plasmonic fields of noble metal nanostructures when the analyte is in their close proximity or placed in the so-called *hot spots*, nanogaps between optically coupled nanoparticles where electromagnetic field enhancement can reach very intense values [14,15]. This enhancement enables the identification of molecular vibrations with high sensitivity, even when only minimal sample amounts are available down to single molecule [16]. It is worth noting that the challenge of this approach lies in the identification of allergens in complex matrices such as pollen or food, or in the detection of their antigens in bodily fluids (e.g., blood or saliva). The growing development of spectroscopic analysis methodologies using artificial intelligence [17,18,19] will play a pivotal role in the future application of these spectroscopic techniques, which makes the development of increasingly comprehensive and accurate databases a crucial point.

**Figure 1:**
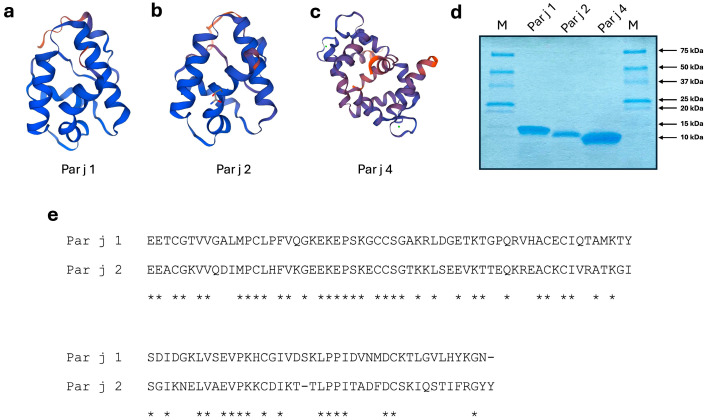
(**a**–**c**) Backbone ribbon representation of the Par j 1, Par j 2 and Par j 4 allergens, respectively. Models were determined using the services of the Swiss-Model Protein Modelling Server using the PDB entry O04403.1 for the ns-LTPs and the PDB entry 2OPO for the calcium binding protein. (**d**) Coomassie staining of purified recombinant Par j 1, Par j 2 and Par j 4. M line indicates a molecular mass marker. Numbers indicate the size of the marker proteins. (**e**) Alignment of the amino acid sequences of the Par j 1 and Par j 2 allergens. The entries displayed are Par j 1 (#Q40905), Par j 2 (#P55958). Asterisks indicate homologies.

For the above reasons, in this study, we exploited both the Raman and SERS techniques to identify the biomolecular vibrational fingerprints of *Parietaria judaica* allergens, for the first time in the literature. We focused on spectral characterization of the major *Parietaria* allergens, specifically Par j 1 and Par j 2. Additionally, we have used Par j 4 as a model to demonstrate that this technique can effectively differentiate between the two classes of proteins (major and minor *Parietaria* allergens). All Par j allergens (Par j 1, Par j 2 and Par j 4) were produced using recombinant DNA technology [20]. Their backbone ribbon representations are shown in Figure 1a–c. These proteins share similarities with the predominant allergenic epitopes present in *Parietaria judaica* pollen, suggesting that their examination could signify a notable step forward in both molecular diagnosis and the creation of personalized vaccines [20]. Exploiting the SERS effect from a silver dendrite substrate [21] allowed us to highlight the spectral differences between the three allergens, in line with the amino acid abundance in their primary structure.

This approach represents an innovative platform enabling the precise identification and differentiation of various proteins, thereby facilitating faster and more accurate allergy diagnoses.

## 2. Materials and Methods

### 2.1. Production of Recombinant Allergenic Proteins

Recombinant *Parietaria* allergens were expressed as His-tagged fusion protein using the pQE30 expression vector (QIAGEN, Milano, Italy) as previously described [22]. Briefly, the recombinant proteins were purified in a single purification step by means of a His Trap column (GE, Uppsala, Sweden) following the manufacturer’s instructions. The purity and concentration of the proteins were determined by Coomassie brilliant blue staining and densitometric analysis, respectively (Odissey Quantity ONE Software 3.0, LI-COR, Lincoln, NE, USA) (see Figure 1d for details). The buffer phosphate saline (PBS) solution at pH 7.4, made of potassium phosphate monobasic (KH_2_PO_4_), sodium chloride (NaCl) and sodium phosphate dibasic (Na_2_HPO_4_·7H_2_O), was purchased by Thermo-Fisher Scientific, Monza, Italy (catalog number 10010023). Using this method, we obtained 1 µg/µL for the Par j 1 protein and 3 µg/µL for the Par j 2 and 4 proteins, which were the maximum achievable amounts without observing aggregation or precipitation phenomena. These protein amounts were then adopted for both Raman and SERS experiments.

### 2.2. Ag Dendrites Synthesis and SERS Sample Preparation and Characterization

Ag dendrites were produced using a metal-assisted chemical etching (MACE) approach with silver salts [23]. To fabricate Ag fractals, we used 4-inch (100)-silicon commercial wafer with a 500 μm thickness obtained from Siegert Wafer (Charlottenburger Allee 7, 52068 Aachen, Germany). An aqueous 2.5 M hydrofluoric acid (HF) solution (Sigma-Aldrich—Merck, Milano, Italy) was utilized to remove the native oxide. The watery etching solution to carry out the MACE process comprised 0.02 M silver nitrate (AgNO_3_, by Scharlau—Scharlau Turkey, Solen Residence A Blok No: 19/4 Ic Kapi No: 109 Tasocagi Yolu Cad. Mahmutbey, Istanbul, Turkey) and 5 M HF. The UV–ozone treatment processes were carried out using an “Ossila” UV–ozone cleaner (Sheffield, UK).

Silver fractals were characterized in cross-section view by scanning electron microscopy using a ZEISS Supra 25 instrument (Carl-Zeiss-Straße 22 73447 Oberkochen, Germany). Extinction spectroscopy measurements were performed using a spectrophotometer (Perkin Elmer, Waltham 940 Winter St, Waltham, MA, USA) equipped with an integrating sphere in diffuse reflectance configuration mode. The transmission configuration was not feasible because the silicon substrate significantly absorbs light in the visible range. Consequently, the reflectance spectrum was transformed into apparent absorbance (extinction), calculated as the logarithm of 100 divided by the reflectance percentage (log(100/R%)).

### 2.3. Raman and SERS Measurements

The Raman measurements were performed using a Raman-grade CaF_2_ commercial slide as a support substrate to avoid any spurious fluorescence contribution, characterized by a very low amount of Raman peaks [19,24]. Raman and SERS spectra were acquired using a HORIBA iHR550 spectrometer (Horiba, Kyoto, Japan) with a 600 lines/mm grating. The spectrometer is connected to an Olympus microscope BX51 equipped with a Super-Head for selecting the 473 nm laser line (solid-state COBOLT—HÜBNER Group, Kassel, Germany). Measurements were performed by focusing 5 mW (for Raman experiments) and 45 µW (for SERS experiments) through a 100× objective with a numerical aperture (NA) of 0.9. In the Raman experiments, each protein solution was deposited via drop casting onto a CaF_2_ substrate and analyzed, first by focusing directly on the liquid drop, and then on the solid residue left to dry. In this latter case, the protein concentrates on the CaF_2_ substrate, depending on its low wettability. Measurements were performed at the *coffee ring*, where the material was most concentrated, aiming to increase the scattering volume. In contrast, for the SERS measurements, the solution was applied to the percolative plasmonic substrate, where it spread and diffused. SERS analyses were carried out within ten minutes of drop-casting, with no visible meniscus in any case. The backscattered signals were collected over 10 s (Raman) and 5 s (SERS) and detected using a Peltier cooled Syncerity CCD (HORIBA). All spectra were recorded in a range between 100 cm^–1^ and 1800 cm^–1^ to capture the full spectrum of typical vibrational modes of proteins. Both raw Raman and SERS spectra were processed using LabSpec 6 software from Horiba. First, the fluorescence contributions were removed by interpolating straight lines between selected reference points on the spectra (<15 between 100 and 4000 cm^−1^), and then they were smoothed with a Savitzky–Golay filter, applying a linear regression over successive sub-sets of 9 adjacent data points.

## 3. Results and Discussion

*Parietaria* allergens (known also as pellitory or wall pellitory) primarily consist of two major proteins: Par j 1 and Par j 2, belonging to the nonspecific lipid transfer protein (ns-LTP) family which represents one of the major classes of allergenic proteins with particular reference to food allergens [20]. They are both glycoproteins, capable of causing severe allergic reactions due to their 3D structure composed of four alpha-helices forming a hydrophobic cavity. This region can bind and transfer lipid molecules and it is stabilized by the presence of eight cysteines, forming four cysteine bridges with a signature characteristic of all the components of the ns-LTP allergens [6]. This class of allergens is known for its stability and resistance to degradation, allowing it to remain active and allergenic for prolonged periods. Par j 1 and Par j 2 display a 51.5% homology at the amino acid level, as shown in Figure 1e. Both proteins are cross-reactive, but the subtle differences in their structure can lead to variations in the immune response of different individuals [5]. So far, no cross-reactivity with other ns-LTP from other sources has been described. Calcium-binding proteins (CBP) contain a variable number of motifs, termed EF-hands, which consist of two perpendicularly placed alpha-helices and an inter-helical loop forming a single calcium-binding site. Par j 4 is a cross-reactive 2-EF-hand allergen CBP which is a minor allergen in the Mediterranean area. It has been clearly demonstrated its cross reactivity with other components of the CBP family from several allergenic sources [25]. Par j 1–2 and Par j 4 do not display any relevant homology at the amino acid level and do not show immunoglobulin E (IgE) cross reactivity [25]. In Table S1 of the Supplementary Materials, the abundance of amino acids, different for each analyzed Par j protein, is shown.

**Figure 2:**
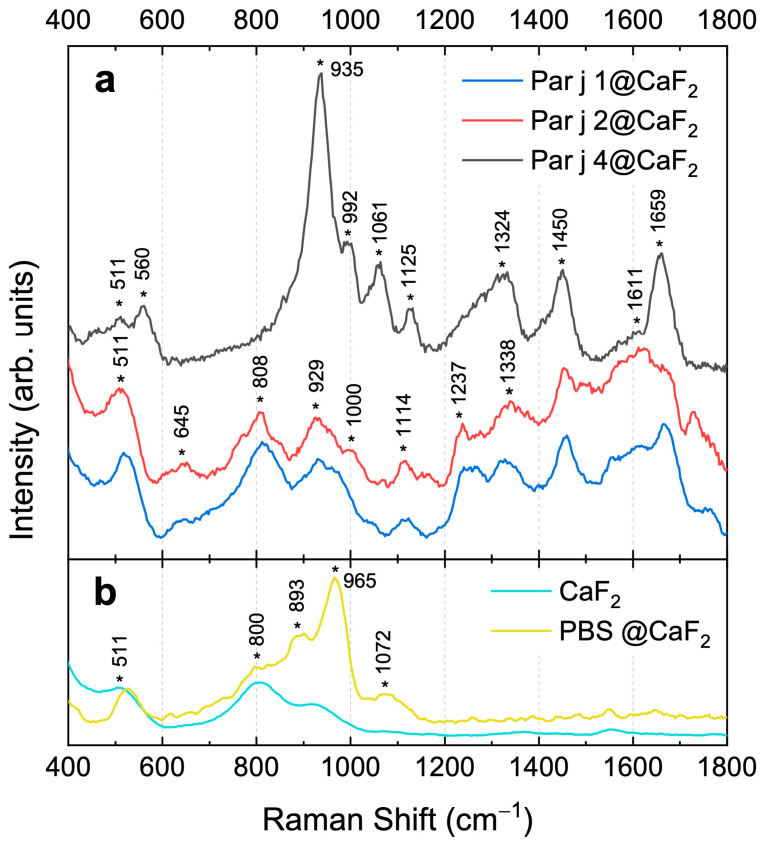
(**a**) Raman spectra of Par j 1 (blue line), Par j 2 (red line), and Par j 4 (black line) acquired on CaF_2_ substrate. The spectra are stacked for a better visualization. (**b**) Raman spectrum of PBS on CaF_2_ substrate (yellow line) and that of CaF_2_ substrate itself (cyan line). Asterisks * indicate the center frequencies of the Raman peaks.

The three recombinant proteins of *Parietaria judaica* have been characterized by Raman spectroscopy [26,27,28,29,30] for the first time in the literature. Raman spectroscopic analysis was initially performed on protein solutions in PBS (see Section 2.3). However, when acquiring Raman signals from the liquid drop, only the O-H bending and stretching modes of water were detected, as the protein’s vibrational signals remained below the detection threshold (see Figure S1 in the Supplementary Information). To address this limitation, we attempted to concentrate the protein on the substrate, although this resulted in the loss of information regarding the number of molecules per unit volume. Figure 2 shows the Raman spectra in the region between 400 and 1800 cm^−1^ representing the protein fingerprint. All spectra were acquired by exciting the protein onto a CaF_2_ substrate using a wavelength of 473 nm, as described in Section 2.3. The top panel (a) of the graph shows the spectra of the three proteins (Par j 1, blue line, Par j 2, red line, and Par j 4, black line) after removing the fluorescence contribution (see Figure S2 in Supplementary Information for details), while the bottom panel (b) shows the Raman spectra of the reference materials, i.e., the CaF_2_ substrate (cyan line) and the PBS deposited on it (yellow line). The protein fingerprints of Par j 1 and Par j 2 are very similar, confirming the analogies in their primary structure (see Figure 1). Table 1 provides the assignments of the main vibrational contributions highlighted in the graph. Signals at 645 cm^−1^, 1000 cm^−1^, 1114 cm^−1^, 1237 cm^−1^, 1338 cm^−1^, 1450 cm^−1^, 1611 cm^−1^, and 1659 cm^−1^ are attributable to the vibrational modes of protein functional groups (Table 1). In contrast, signals at 511 cm^−1^, 808 cm^−1^, and 929 cm⁻¹ are challenging to discriminate, being probably due to the overlapping between the protein vibrational contributions with signals coming from CaF_2_ and PBS (see Figure 2b).

The Raman spectrum of Par j 4 exhibits significant differences from those of Par j 1 and Par j 2, due to variations in its amino acid sequence [25]. Par j 4 does not show any fluorescence background, resulting in a much more resolved and intense Raman spectrum. Despite the structural differences between Par j 4, and Par j 1 and 2, many vibrational modes are common in all spectra (see Table 1).

In the Raman experiment, each protein solution was deposited onto a CaF_2_ substrate and left dry. The measurements were conducted onto the *coffee ring*, where most of the material had concentrated, thereby enhancing the Raman scattering volume and consequently its signal. Despite this approach, some key vibrational contributions useful for distinguishing the three proteins exhibit intensities that are too low and are obscured by the signal from PBS. To overcome this issue, we adopted a specific SERS approach using a SERS platform made of Ag dendrites. This plasmonic material is obtained as a by-product of the silicon nanowire (Si NW) synthesis by the metal-assisted chemical etching (MACE) technique [23,33,34], as previously described in the Materials and Methods Section (Section 2.2). This chemical procedure relies on high electronegative metals such as silver and gold to catalyze and drive the silicon oxidation and etching process [34,35,36,37]. As a result, a network of silver dendrites is formed on top of the silicon substrate previously immersed inside the chemical solution. The sample is then left to dry inside a chemical fume hood for more than 12 h.

The final samples for SERS measurements were prepared by initially cleaning the silver dendrites with 2 min of UV–ozone treatment to remove any possible organic contamination. This process successfully removes organic contaminants from the surface, as shown in Figure S3 of the Supplementary Information, thus eliminating any undesired signal that would affect the SERS signal from the biomolecules. Subsequently, for each protein, 10 µL of solution was drop-cast onto the Ag substate.

**Figure 3:**
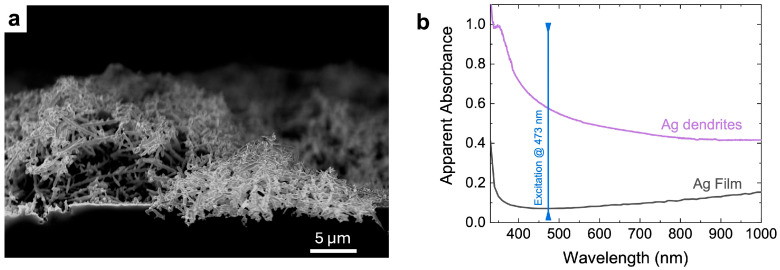
(**a**) Scanning electron microscopy image of the as-grown Ag dendrites in plan view; (**b**) apparent absorbance (extinction) spectrum of Ag dendrites (violet line) and Ag flat substrate (black line) [39,40] in the UV–vis–NIR range, obtained as commented in Section 2.2.

The dendrites appear as an intriguing material about 15 microns thick; they rest randomly distributed on silicon nanowires (see Figure 3a), thus creating a plasmonic layer with peculiar characteristics. Figure 3b shows a comparison of the optical properties of the Ag fractal network (violet spectrum), measured using UV–VIS spectroscopy, with those of a continuous Ag bulk layer (black spectrum) used as a reference from the literature [21]. In particular, the apparent absorbances of the two materials, obtained from the reflectance (R%) as log(100/R%) are plotted in the range between 310 and 1000 nm. In the case of Ag dendrites, a very broad plasmon resonance peak extends throughout the whole visible range. This broadening of the whole wavelength range results from the intricate morphology of the Ag nanostructures. Their fractal nature, indeed, ensures the optical coupling with a wide range of exciting wavelengths spanning from the near UV to the near infrared as well as the generation of intense *hot spot* regions. This occurrence promotes a calculated SERS enhancement factor (EF) exceeding 10^6^ when the excitation wavelength was set at 633.8 nm [21,38]. The shape of the apparent absorbance (extinction) spectrum, depicted in Figure 3b, could suggest an EF slight progressive increase towards the blue spectral region that is hard to demonstrate here, however, since the Raman signals of allergen in liquid drops are below the detection threshold, as already commented.

Due to their unique 3D structure with micro- and nanocavities and their fractal nature, silver dendrites serve as an optimal SERS platform enabling the analysis of molecules in hydration conditions, the physiological environment of biomolecules, even several hours after their introduction into them (see Figure S4 in Supplementary Information for details and comments). Similar to a sponge, indeed, this material entraps biomolecules within very small cavities in a liquid environment [21,38]. The interplays between the fractal metal nanostructures and the exciting electromagnetic field generates highly efficient *hot spots* into these nanocavities, allowing for extremely sensitive spectroscopic sensors operating in the natural habitat of biomolecules.

**Figure 4:**
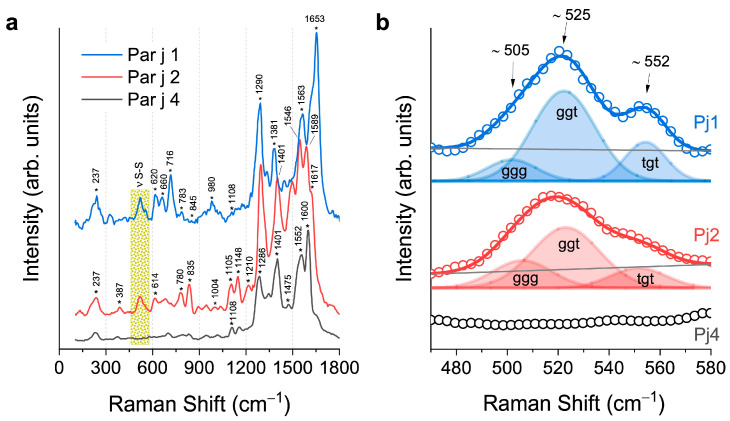
(**a**) SERS spectra of Par j 1 (blue line), Par j 2 (red line), and Par j 4 (black line) acquired onto the Ag fractal. Asterisks * indicate the center frequencies of the Raman peaks. (**b**) Comparison and fit of the S–S stretching bands of the proteins in the low Raman shift region. For a better visualization, the spectra are stacked in each graph.

Figure 4a displays the SERS spectra acquired for each recombined protein Par j 1 (blue line), Par j 2 (red line), and Par j 4 (black line), obtained after removing the fluorescence background (see Figure S5). The Raman signal with the lowest frequency, located at 237 cm^–1^, is ascribed to the Ag–O stretching of the silver dendrite substrate, which corresponds to the sole non-protein signal observed in the spectra (see Figure S3 for details). Instead, the higher frequency signals are all associated with the vibrational modes of the protein functional groups.

The region between 400 cm^–1^ and 1800 cm^–1^ is characterized by the typical protein signals. Starting from the lower Raman frequencies, the disulfide bridges’ vibrational modes are detectable in the range from 470 to 580 cm^−1^, as commented in detail below. The primary structure of the Par j contains the most prevalent amino acids, whose abundance across the three proteins affects the Raman vibrational spectra (see Table S1, in Supplementary Materials). Specifically, in the Par j 1 protein, vibrational modes mainly associated with lysine (783 cm^–1^), and histidine (980 cm^–1^) are detected and identified. In the Par j 2 spectra, lysine’s contribution is notably evident in the signals centered at 780 cm^–1^, 1148 cm^–1^, and 1210 cm^–1^. Despite Par j 1 and 2 having a very similar primary structure, as detected by their comparable Raman spectra (see Figure 2), the differing lysine abundance in their sequences (Table S1) aligns perfectly with the variations in the intensity of the SERS peaks between the two allergens, providing a key distinguishing feature in their vibrational fingerprints. In Par j 4, the predominant contributions of alanine, arginine, and serine are revealed in the range between 860 cm^–1^ and 1120 cm^–1^. It is also evident that the SERS spectra in Figure 4 differ from the Raman spectra in Figure 2, as only the former undergo selective amplification of certain vibrational modes. This amplification is influenced by the orientation of the molecules relative to the plasmonic substrate and their ability to interact with it. Consequently, the intensity ratios in the SERS spectrum often vary significantly from those in the Raman spectrum, as demonstrated in this case. A statistical analysis, presented in Figure S6 of the Supplementary Information, was conducted on five points for each Par j protein at the same experimental conditions mentioned here.

In Table 2, the previous assignments of the Raman peaks are reported in detail. The breathing mode of the phenylalanine (Phe) aromatic ring can be identified at 1004 cm^−1^, although it is not always well visible, probably due to the limited numbers of Phe units (approximately 3–5 per protein), which are insufficient to generate a considerable signal across all protein conformations. The deformation modes of CH and CH_2_ groups, such as the twisting, bending, and rocking vibrations between 1300 cm^–1^ and 1330 cm^–1^, and the CH_2_ scissoring modes between 1430 cm^–1^ and 1480 cm^–1^, are found to be intense in this spectral region. On the other hand, the complex vibrational modes present in the peptide backbone, commonly known as amides, display contributions across different frequencies, though with lower intensities compared to side-chain residues that are closely exposed to the metal surface and thus more susceptible to plasmonic enhancement. The amide III band, deriving by the coupled C–N stretching and N–H bending [32] is well visible between 1220 cm^–1^ and 1400 cm^–1^. The amide II band, originating from N–H vibrations and C–N stretching, is found in the range between 1480 cm^–1^ and 1580 cm^–1^. Finally, the amide I band, arising from the C=O stretching vibrations and the out-of-plane C–N stretching, is detected within the range of 1600 cm^–1^ to 1660 cm^–1^ [41].

Furthermore, Raman spectroscopy via SERS spectra was an efficient tool in recognizing Par j 4 with respect to Par j 1 and 2 through the analysis of S-S disulfide bonds peaks of cysteine. This spectral region is highlighted by the yellow rectangle. Par j 4 protein is characterized by the absence of the cysteine in the amino acid sequence, unlike the case of Par j 1 and 2 (see Table S1). Free cysteine, with S-H residues, oxidizes to form disulfide bonds characterized by the S-S atomic group. Figure 4b shows a magnification of it, in the range between 460 cm^–1^ and 580 cm^–1^. Par j 1 and 2 exhibit a large, structured band between 470 cm^–1^ and 580 cm^–1^. Different Raman vibrations lie in this spectral range as the frequency of the disulfide bond is very sensitive to the conformation of the CC-S-S-CC functional group in the protein. Therefore, in Figure 4b, we propose the interpretation of the detected Raman bands, based on the model of disulfide bonds, whose deconvolution and analysis through fitting reveal different conformational contributions: the gauche-gauche-gauche (*ggg*) at 508 cm^–1^, the gauche-gauche-trans (*ggt*) at 520 cm^–1^, and the trans-gauche-trans (*tgt*) at 544 cm^–1^ [32,42]. In Par j 2, the *ggg* contribution is barely visible as a low-frequency shoulder of the main *ggt* band. However, it is worth noting that within this range, the Si–Si stretching contribution of the Si NW substrate at 520 cm^–1^, whose visibility relies on the depth of laser excitation focus within the 3D structure, can overlap with the disulfide bridge band, thereby altering the intensity ratio between the bands. Moreover, the presence of vibrational contributions from Ile and Thr could manifest in Raman spectra around 560 cm^–1^, affecting the shape of the disulfide bridge band on the tail (see Table S1 regarding the amino acid abundance and Table 1 for the Raman assignments). The Par j 4 spectrum is shown in black for comparison; clearly, no disulfide bridge Raman modes are present in this region as cysteine is absent in the protein, but also in this case, the vibrational contributions of Ile and Thr could be detected. In conclusion, the method employed may facilitate the identification and distinction of allergens, aligning with the amino acid composition in their primary structure (see Table S1).

## 4. Conclusions

In this study, we revealed the first Raman fingerprints of three protein allergens of the *Parietaria judaica* pollen (Par j 1, Par j 2, and Par j 4) obtained through recombinant DNA methods. By leveraging the significant SERS enhancement from a dense array of silver dendrites, we obtained a much more detailed fingerprint. Although these proteins originate from the pollen of the same plant, they exhibit dissimilarities that can affect their allergenic reactivity. Recognizing these differences is crucial for accurate diagnosis and the development of effective treatments for allergies caused by *Parietaria judaica* pollen and for the designing of preventive strategies and refining public health measures to minimize allergen exposure. The approach used here enabled the identification and differentiation of the proteins in agreement with the amino acid abundance in their primary structure.

It is important to emphasize that identifying allergens through Raman spectroscopy or SERS in complex matrices like pollen or food, or detecting their antigens in bodily fluids (e.g., blood or saliva), is an extremely challenging task. However, future advancements in artificial intelligence methodologies, still in development, could greatly enhance this process. Additionally, with the aim of functionalizing the plasmonic platform for SERS sensor applications, a thorough understanding of the Raman spectrum of a biomolecule used as a probe would be greatly beneficial, enabling the precise analysis of target–probe interactions.

## Figures and Tables

**Table 1:** Raman peak assignment of *Parietaria judaica* proteins in PBS solution deposited and dried onto CaF_2_ substrate.

Frequency (cm^–1^)	Mode Assignment	Reference	Protein
511	CaF_2_		Par j 1, 2, 4
560	Ile, Thr	[31]	Par j 4
645	OCN bending NH bending out-of-plane	[32]	Par j 1, 2
808	CaF_2_		Par j 1, 2
929–935	C-C stretching CaF_2_, PBS	[15]	Par j 1, 2, 4
1000	Phe ring (C-C) breathing mode	[10,15,31,32]	Par j 1, 2
1061	C-C and CN stretching Lys (C wagging)	[31]	Par j 4
1114–1125	CN stretching	[15]	Par j 1, 2, 4
1237–1338	CH_2_ twisting, CH bending Amide III	[15,31,32]	Par j 1, 2, 4
1450	CH_2_ and CH_3_ scissoring	[31]	Par j 1, 2, 4
1611	Phe Ring stretching	[10]	Par j 1, 2, 4
1659	Amide I	[31]	Par j 1, 2, 4

**Table 2:** Vibrational peak assignment of *Parietaria judaica* proteins by SERS spectra.

*Parietaria judaica* 1		
Frequency (cm^–1^)	Mode Assignment	
237	Ag-O Str.	
508–557	S-S str.(disulfide bonds)	[31,32]
520	Si-Si sym str,	
620	Phe COO- deformation	[31]
625–800	ring (C-C) deformation, OCN bend, NH bend out-of-plane	[31,32]
715–730	Tyr	[15,31]
783	Lys	[15]
845	Leu Lys (Cγ twist., Cε wagg., Cβ wagg.)	[15]
850	Val His (C-H out of plane) Leu Ala Nt–Cα; O–Ct–O; Cα–Ct–O	[15]
897	CC stretching or CCN_sym_ stretching	[31]
931	Ile Arg (Nη1–Cζ–Nη2 sym st; Cδ–Nε; Cβ–Cγ; Nε–Cζ)	[15]
965	CC stretching or CCN_sym_ stretching	[31]
982	His Lys	[15]
1010	Phe ring (C-C) breathing	[10,15,31,32]
1108	His (NtH_3_^+^ asym. rock.) Thr	[15]
1144	Lys (NtH_3_^+^ asym rock; Ct Cα-Hα)	[15]
1172	CN str	[31]
1268–1400	Amide III	[15,31]
1316	CH_2_ twst., CH bend.; His (NtH_3_^+^ asym. rock, Cβ-rock., Ct-Cα-H)	[15,31]
1330	CH_2_ twst., CH bend.	[31]
1438–1467	CH_2_ and CH_3_ scissoring	[31]
1480–1580	Amide II	[15,32]
1600–1660	Amide I	[31]
***Parietaria judaica*** **2**		
**Frequency** **(cm^–1^)**	**Mode Assignment**	
237	Ag-O Str.	
508–557	S-S str.(disulfide bonds)	[31,32]
630–800	OCN bend, NH bend out-of-plane	[32]
780	Lys	[15]
835	Phe, Tyr	[15]
890–980	CC stretching or CN_sym_ stretching	[32]
1085	CN str.	[31]
1124	Val, Ser	[15]
1148	His Lys (NtH_3_^+^ asym rock; Ct Cα-Hα)	[15]
1162	Lys	[15]
1210	Lys, Tyr, Phe	[15,31]
1220–1400	Amide III	[32]
1320	CH_2_ twst., CH bend.; His (NtH_3_^+^ asym. rock, Cβ-rock., Ct-Cα-H)	[15,31]
1340	CH_2_ twst., CH bend.	[31]
1386	Cys	[15]
1401	His (Nt–Cα–Hα, Cβ–Cα–H, Cβ-rock)	[15]
1480–1580	Amide II	[15,32]
1589	Phe	[31]
1600	Tyr + Phe ring stretching	[31]
1617	Phe ring stretching	[10]
1600–1660	Amide I	[31]
***Parietaria judaica*** **4**		
**Frequency** **(cm^–1^)**	**Mode Assignment**	
237	Ag-O Str.	
792	NH bend out-of-plane	[32]
810	Ser, Met	[15]
835	Phe	[15]
860	Ala (Nt–Cα; O–Ct–O; Cα–Ct–O sym bend; Cα–Ct) Arg (Cζ–Nη2) Ser	[15]
910–950	CC stretching or CN_sym_ stretching	[31,32]
1004	Phe ring (C-C) breathing	[10,15,31,32]
1100	NH_3_^+^ rocking Lys (Cε–Nζ; Cı–Cε) Arg (Cγ–Cδ) Gly (N–Cα)	[15,31]
1225–1275	Amide III	[31,32]
1300–1350	CH def., CH_2_ twst., CH bend.	[15,32]
1401	His (Nt–Cα–Hα, Cβ–Cα–H, Cβ-rock)	[15]
1475	CH_2_ and CH_3_ scissoring	[31,32]
1482–1533	Amide II	[15,32]
1552	Amide II	[15]
1600–1660	Amide I	[15]

Ct and Nt refer to carbon (C) and nitrogen (N) of the terminal COO^−^ and NH_3_^+^ groups, respectively [31].

## Data Availability

The raw data supporting the conclusions of this article will be made available by the authors on request.

## Supplementary Materials

The following supporting information can be downloaded at: https://www.mdpi.com/article/10.3390/bios15030182/s1, Figure S1: Raman spectra of Par j proteins in PBS solutions; Figure S2: Raw Raman spectra; Figure S3: Ag dendrite Raman spectra before and after the UV-O_3_ treatment; Figure S4: SERS spectra in the high-frequency Raman region; Figure S5: Raw SERS spectra; Figure S6: Reproducibility of SERS spectra; Table S1: Amino acid abundance.

## Abbreviations

The following abbreviations are used in this manuscript:

Par j 1	*Parietaria judaica* 1
Par j 2	*Parietaria judaica* 2
Par j 4	*Parietaria judaica* 4
SERS	Surface-enhanced Raman spectroscopy

## References

[1] Ishizaka K., Ishizaka T. (1967). Identification of γE-Antibodies as a Carrier of Reaginic Activity. J. Immunol..

[2] Hu J., Chen J., Ye L., Cai Z., Sun J., Ji K. (2018). Anti-IgE Therapy for IgE-Mediated Allergic Diseases: From Neutralizing IgE Antibodies to Eliminating IgE^+^ B Cells. Clin. Transl. Allergy.

[3] Izmailovich M., Semenova Y., Abdushukurova G., Mukhamejanova A., Dyussupova A., Faizova R., Gazaliyeva M., Akhvlediani L., Glushkova N., Kalmakhanov S. (2023). Molecular Aspects of Allergen-Specific Immunotherapy in Patients with Seasonal Allergic Rhinitis. Cells.

[4] Ciprandi G., Puccinelli P., Incorvaia C., Masieri S. (2018). Parietaria Allergy: An Intriguing Challenge for the Allergist. Medicina.

[5] Colombo P., Bonura A., Costa M.A., Izzo V., Passantino R., Locorotondo G., Amoroso S., Geraci D. (2003). The Allergens of Parietaria. Int. Arch. Allergy Immunol..

[6] Colombo P., Kennedy D., Ramsdale T., Costa M.A., Duro G., Izzo V., Salvadori S., Guerrini R., Cocchiara R., Mirisola M.G. (1998). Identification of an Immunodominant IgE Epitope of the *Parietaria judaica* Major Allergen. J. Immunol..

[7] Bonura A., Gulino L., Trapani A., Di Felice G., Tinghino R., Amoroso S., Geraci D., Valenta R., Westritschnig K., Scala E. (2008). Isolation, Expression and Immunological Characterization of a Calcium-Binding Protein from *Parietaria* Pollen. Mol. Immunol..

[8] Molecular Allergology User’s Guide 2.0. https://hub.eaaci.org/resources_documents/molecular-allergology-users-guide-2-0/.

[9] Chen M.C., Lord R.C. (1976). Laser-Excited Raman Spectroscopy of Biomolecules. VIII. Conformational Study of Bovine Serum Albumin. J. Am. Chem. Soc..

[10] David C., d’Andrea C., Lancelot E., Bochterle J., Guillot N., Fazio B., Maragò O.M., Sutton A., Charnaux N., Neubrech F. (2012). Raman and IR Spectroscopy of Manganese Superoxide Dismutase, a Pathology Biomarker. Vib. Spectrosc..

[11] Moskovits M. (1985). Surface-Enhanced Spectroscopy. Rev. Mod. Phys..

[12] Ru E.L., Etchegoin P. (2008). Principles of Surface-Enhanced Raman Spectroscopy: And Related Plasmonic Effects.

[13] Lo Faro M.J., Leonardi A.A., Morganti D., Sciuto E.L., Irrera A., Fazio B. (2022). Surface-Enhanced Raman Scattering for Biosensing Platforms: A Review. Radiat. Eff. Defects Solids.

[14] Nie S., Emory S.R. (1997). Probing Single Molecules and Single Nanoparticles by Surface-Enhanced Raman Scattering. Science.

[15] Fazio B., D’Andrea C., Foti A., Messina E., Irrera A., Donato M.G., Villari V., Micali N., Maragò O.M., Gucciardi P.G. (2016). SERS Detection of Biomolecules at Physiological pH via Aggregation of Gold Nanorods Mediated by Optical Forces and Plasmonic Heating. Sci. Rep..

[16] Kneipp K., Wang Y., Kneipp H., Perelman L.T., Itzkan I., Dasari R.R., Feld M.S. (1997). Single Molecule Detection Using Surface-Enhanced Raman Scattering (SERS). Phys. Rev. Lett..

[17] D’Andrea C., Cazzaniga F.A., Bistaffa E., Barucci A., de Angelis M., Banchelli M., Farnesi E., Polykretis P., Marzi C., Indaco A. (2023). Impact of Seed Amplification Assay and Surface-Enhanced Raman Spectroscopy Combined Approach on the Clinical Diagnosis of Alzheimer’s Disease. Transl. Neurodegener..

[18] Srivastava S., Wang W., Zhou W., Jin M., Vikesland P.J. (2024). Machine Learning-Assisted Surface-Enhanced Raman Spectroscopy Detection for Environmental Applications: A Review. Environ. Sci. Technol..

[19] Morganti D., Rizzo M.G., Spata M.O., Guglielmino S., Fazio B., Battiato S., Conoci S. (2024). Temporal Convolutional Network on Raman Shift for Human Osteoblast Cells Fingerprint Analysis. Intell.-Based Med..

[20] Dorofeeva Y., Colombo P., Blanca M., Mari A., Khanferyan R., Valenta R., Focke-Tejkl M. (2019). Expression and Characterization of Recombinant Par j 1 and Par j 2 Resembling the Allergenic Epitopes of *Parietaria judaica* Pollen. Sci. Rep..

[21] Faro M.J.L., D’andrea C., Leonardi A.A., Morganti D., Irrera A., Fazio B. (2019). Fractal Silver Dendrites as 3D SERS Platform for Highly Sensitive Detection of Biomolecules in Hydration Conditions. Nanomaterials.

[22] Licciardi M., Montana G., Bondì M.L., Bonura A., Scialabba C., Melis M., Fiorica C., Giammona G., Colombo P. (2014). An Allergen-Polymeric Nanoaggregate as a New Tool for Allergy Vaccination. Int. J. Pharm..

[23] Chen C.-Y., Hsiao P.-H. (2015). Silver-Assisted Chemical Etching on Silicon with Polyvinylpyrrolidone-Mediated Formation of Silver Dendrites. ChemPhysChem.

[24] Calabrese G., De Luca G., Franco D., Morganti D., Rizzo M.G., Bonavita A., Neri G., Fazio E., Neri F., Fazio B. (2023). Structural and Antibacterial Studies of Novel ZnO and ZnxMn(1−x)O Nanostructured Titanium Scaffolds for Biomedical Applications. Biomater. Adv..

[25] Stumvoll S., Westritschnig K., Lidholm J., Spitzauer S., Colombo P., Duro G., Kraft D., Geraci D., Valenta R. (2003). Identification of Cross-Reactive and Genuine *Parietaria judaica* Pollen Allergens. J. Allergy Clin. Immunol..

[26] Rygula A., Majzner K., Marzec K.M., Kaczor A., Pilarczyk M., Baranska M. (2013). Raman Spectroscopy of Proteins: A Review. J. Raman Spectrosc..

[27] Jenkins A.L., Larsen R.A., Williams T.B. (2005). Characterization of Amino Acids Using Raman Spectroscopy. Spectrochim. Acta Part A Mol. Biomol. Spectrosc..

[28] Lo Faro M.J., Ruello G., Leonardi A.A., Morganti D., Irrera A., Priolo F., Gigan S., Volpe G., Fazio B. (2021). Visualization of Directional Beaming of Weakly Localized Raman from a Random Network of Silicon Nanowires. Adv. Sci..

[29] Benevides J.M., Overman S.A., Thomas G.J. (2003). Raman Spectroscopy of Proteins. Curr. Protoc. Protein Sci..

[30] Maiti N.C., Apetri M.M., Zagorski M.G., Carey P.R., Anderson V.E. (2004). Raman Spectroscopic Characterization of Secondary Structure in Natively Unfolded Proteins:  α-Synuclein. J. Am. Chem. Soc..

[31] Zhu G., Zhu X., Fan Q., Wan X. (2011). Raman Spectra of Amino Acids and Their Aqueous Solutions. Spectrochim. Acta Part A Mol. Biomol. Spectrosc..

[32] Kuhar N., Sil S., Umapathy S. (2021). Potential of Raman Spectroscopic Techniques to Study Proteins. Spectrochim. Acta Part A Mol. Biomol. Spectrosc..

[33] Kolasinski K.W., Tamarov K., Swanson J.D., Unger B.A., Ernst A.T., Aindow M., Kiviluoto R., Lehto V.-P., Riikonen J. (2020). Injection Metal-Assisted Catalytic Etching (MACE) of Si Powder: Discovery of Low-Load MACE and Pore Distribution Tunability Using Ag, Au, Pd, Pt and Cu Catalysts. Meet. Abstr..

[34] Peng K., Fang H., Hu J., Wu Y., Zhu J., Yan Y., Lee S. (2006). Metal-Particle-Induced, Highly Localized Site-Specific Etching of Si and Formation of Single-Crystalline Si Nanowires in Aqueous Fluoride Solution. Chem. A Eur. J..

[35] Morganti D., Leonardi A.A., Lo Faro M.J., Leonardi G., Salvato G., Fazio B., Musumeci P., Livreri P., Conoci S., Neri G. (2021). Ultrathin Silicon Nanowires for Optical and Electrical Nitrogen Dioxide Detection. Nanomaterials.

[36] Huang Z., Geyer N., Werner P., de Boor J., Gösele U. (2011). Metal-Assisted Chemical Etching of Silicon: A Review. Adv. Mater..

[37] Morganti D., Faro M.J.L., Leonardi A.A., Fazio B., Conoci S., Irrera A. (2022). Luminescent Silicon Nanowires as Novel Sensor for Environmental Air Quality Control. Sensors.

[38] Leonardi A.A., Sciuto E.L., Lo Faro M.J., Morganti D., Midiri A., Spinella C., Conoci S., Irrera A., Fazio B. (2022). Molecular Fingerprinting of the Omicron Variant Genome of SARS-CoV-2 by SERS Spectroscopy. Nanomaterials.

[39] McPeak K.M., Jayanti S.V., Kress S.J.P., Meyer S., Iotti S., Rossinelli A., Norris D.J. (2015). Plasmonic Films Can Easily Be Better: Rules and Recipes. ACS Photonics.

[40] Polyanskiy M.N. (2024). Refractiveindex.Info Database of Optical Constants. Sci. Data.

[41] Baronio C.M., Barth A. (2020). The Amide I Spectrum of Proteins—Optimization of Transition Dipole Coupling Parameters Using Density Functional Theory Calculations. J. Phys. Chem. B.

[42] Van Wart H.E., Lewis A., Scheraga H.A., Saeva F.D. (1973). Disulfide Bond Dihedral Angles from Raman Spectroscopy. Proc. Natl. Acad. Sci. USA.

