# Supplementary material for: First Vibrational Fingerprint of Parietaria judaica Protein via Surface-Enhanced Raman Spectroscopy
Source: Biosensors (Basel). 2025 Mar 13;15(3):182. doi: 10.3390/bios15030182 (PMC11940344; doi:10.3390/bios15030182)
Supplement: Supplementary file 1 [file biosensors-15-00182-s001.zip › biosensors-3474785-supplementary.pdf]

biosensors

MDPI

Supplementary Material

First Vibrational Fingerprint of *Parietaria judaica* Protein via Surface-Enhanced Raman Spectroscopy

Dario Morganti[1,2], Valeria Longo[3], Antonio Alessio Leonardi[4], Alessia Irrera[1], Paolo Colombo[3,*], Barbara Fazio[1,*]

[1]CNR IMM-ME, Institute for Microelectronics and Microsystems, Viale F. S. d'Alcontres 31, I-98166, Messina, Italy.
[2]CNR DSFTM, Department of Physical Sciences and Technologies of Matter, Piazzale Aldo Moro, 7 - 00185 Roma, Italy.
[3]CNR IRIB-PA, Institute for Biomedical Research and Innovation, Via U. La Malfa 153, I-90146. Palermo, Italy
[4]Department of chemical, biological, pharmaceutical, and environmental sciences (ChiBioFarAm), Viale F. S. d'Alcontres 31, I-98166, Messina, Italy
* barbara.fazio@cnr.it (Barbara Fazio); paolo.colombo@irib.cnr.it (Paolo Colombo).

Figure S1: Raman spectra of Par j proteins in PBS solutions

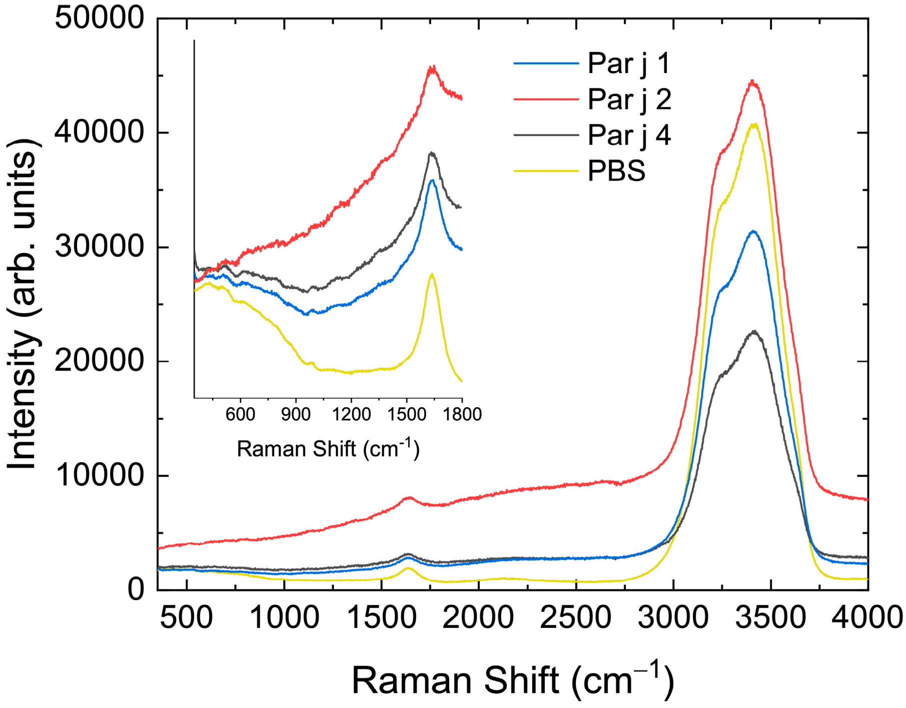

Figure S1. Raman spectra of Par j proteins in PBS solutions at the concentrations of 1 µg/µL for Par j 1 (blue line), and 3 µg/µL for Par j 2 (red line) and Par j 4 (black line). The spectrum of the PBS solution is reported by the yellow line. All the spectra have been acquired for 5 s. In the inset, the same samples acquired for a longer time (60 s) are reported in the protein fingerprint spectral region.

Figure S1 shows the spectra of the Par j 1 (blue line), 2 (red line), and 4 (black line) proteins in PBS solution, alongside the spectrum of PBS (yellow line) for comparison. The sample

solutions have been deposited via drop casting onto CaF_2 substrates and analyzed by focusing the laser onto the liquid drop. Notably, the spectra presented here exclusively exhibit features arising from the vibrational contributions of liquid water. Specifically, the O-H bending mode is observed around 1650 cm^{-1}, while the O-H stretching modes appear in the 3100–3800 cm^{-1} region. When integrating for a longer time (60 s), a peak at 965 cm^{-1} originating from PBS can be observed.

Figure S2: Raw Raman spectra

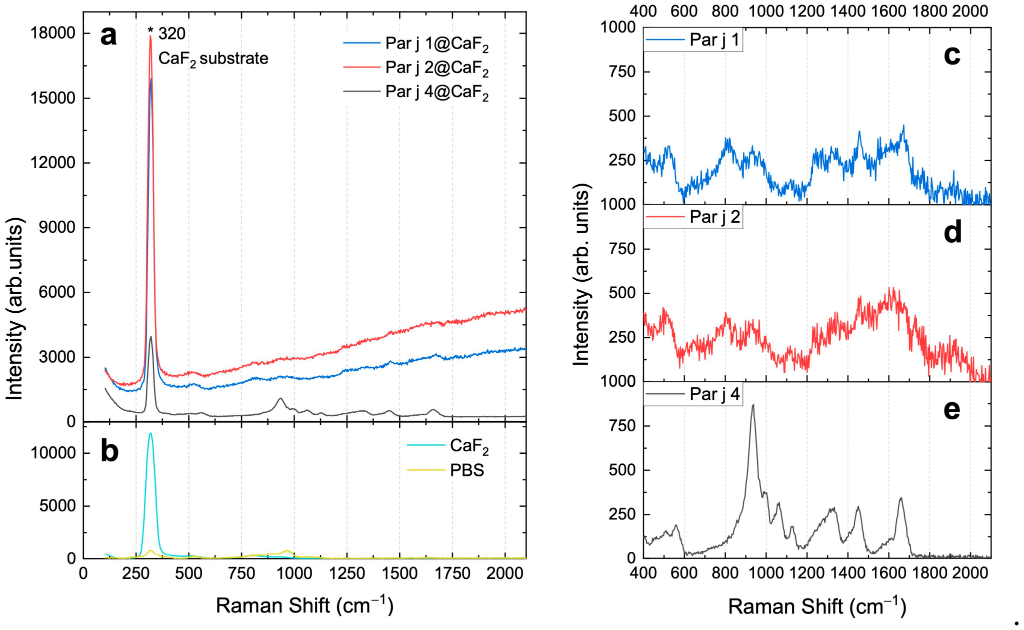

Figure S2. In panel (a) the raw Raman spectra (without the removal of fluorescence background) of Par j 1 (blue line), Par j 2 (red line), Par j 4 (black line) drop-cast onto CaF_2 and left to dry are reported. The yellow line and the cyan line in panel (b) represent the Raman spectrum of PBS solutions dried onto CaF_2 and of the substrate itself, respectively. In panels (c,d and e), the same spectra of Par j 1, 2 and 4 allergens in PBS reported after the fluorescence removal. All the spectra are presented without undergoing a smoothing procedure.

In Figure S2a, the raw Raman spectra of Par j 1 (blue line), Par j 2 (red line) Par j 4 (black line) and PBS (yellow line) acquired on CaF_2 substrate (cyan line, shown as reference) without the removal of the fluorescence contribution are reported. We show all the spectra in the range between 100 cm^{-1} and 1800 cm^{-1}, where the contribution of CaF_2 is clearly visible at 320 cm^{-1}. The spectra of Par j 1 and 2 show fluorescence contributions that cover the already very weak Raman signals. In contrast, the spectrum of Par j 4 shows much more intense vibrational contributions that emerge from a lower fluorescence background compared to the other proteins. Figure S2b presents the raw Raman spectra of the PBS solution dried on a CaF_2 substrate and the bare calcium fluoride substrate, both confirming the absence of a broad fluorescence contribution. In all spectra reported in Figure S2, the prominent first-order Raman peak of CaF_2 at 320 cm^{-1} is clearly visible, while the second-order peaks (400–1000 cm^{-1}) exhibit very low intensities.

The fluorescence signals were removed by subtracting the PL curve attached to the Raman spectrum by using the Horiba LabSpec software. The subtracted spectra are shown in Figure S2c–e for Par j 1 (blue line), Par j 2 (red line), and Par j 4 (black line), respectively.
The signal to noise ratio for Par j 1 and 2 spectra (Figure S2c,d) was calculated according to the following standard equation:

$$S/N = \frac{S_{(1114)} - S_{(2000-2100)}}{N_{\mathrm{rms}}}$$

where $S_{(1114)}$ is the Raman peak intensity at 1114 cm^{-1}, $S_{(2000-2100)}$ is the noise between 2000 and 2100 cm^{-1}, where no Raman signal is present, and N_{rms} is the root mean square of the noise on the background signal.
We obtained a value greater than three for both Par j 1 and 2, whose raw spectra exhibit higher noise levels compared to those of Par j 4. The obtained S/N values provide confidence in the peak attributions.

Figure S3: Ag dendrite Raman spectra before and after the UV-O$_3$ treatment

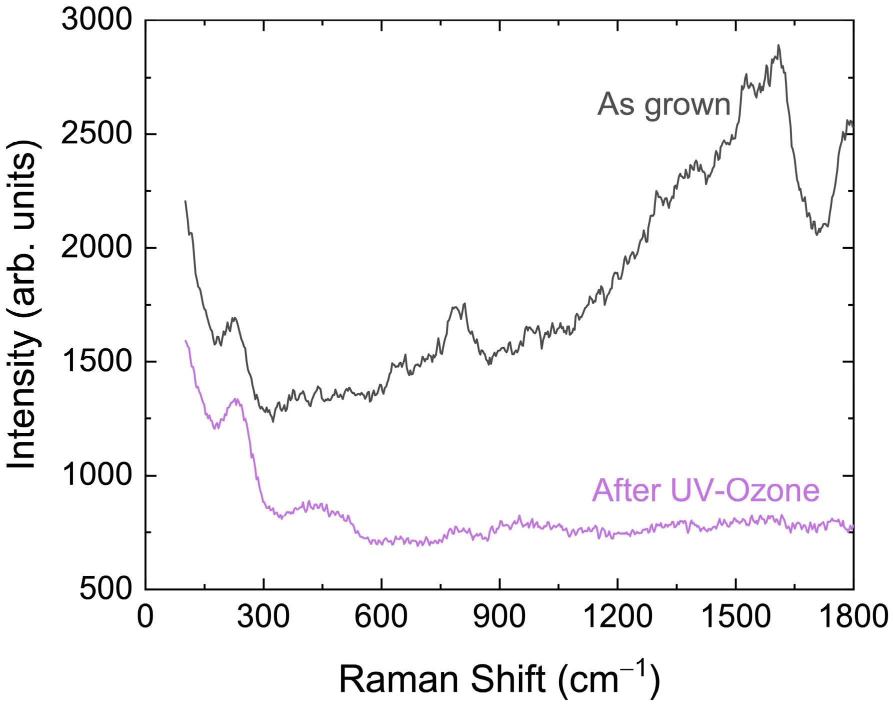

Figure S3. Raman spectra of bare Ag dendrite substrate acquired before (grey) and after (violet) 2 minutes of UV-ozone cleaning process. Both spectra were acquired for 10 seconds, by using an excitation light power of 45 mW through a 100X microscope objective.

In Figure S3, the effectiveness of the UV–ozone cleaning procedure to remove any contamination for the Ag dendrites affecting the Raman signals is demonstrated. Indeed, as it is possible to observe by the comparison of the two Raman spectra, after 2 minutes of UV–ozone, all the Raman signals ascribed to sample contamination became negligible. We used a treatment of 2 minutes as it was the minimum time required to remove any surface contamination affecting the Raman response of the SERS substrate. The only

bands detected correspond to the Ag-O vibrations of the thin oxide layer (237 cm^{-1} and 430 cm^{-1}) on the surface of the silver dendrites, which, however, does not prevent the SERS effect, although it may influence its performance.

Figure S4: SERS spectra in the high-frequency Raman region

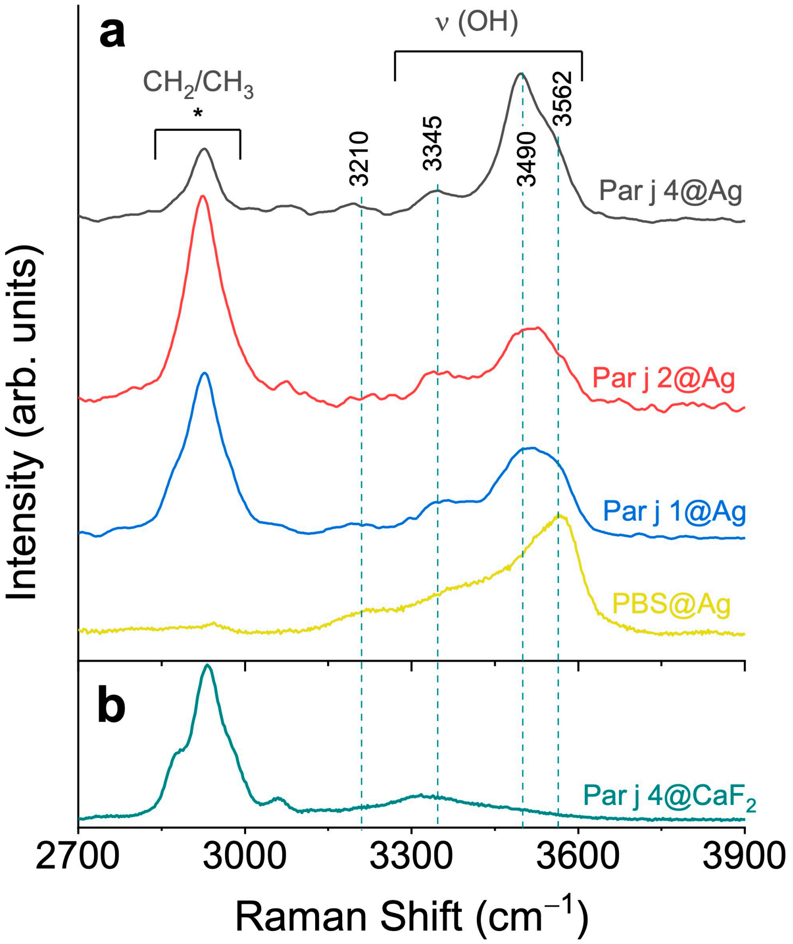

Figure S4. (a) SERS spectra in the high-frequency Raman region of Par j 1 (blue line), Par j 2 (red line), Par j 4 (black line), all in PBS solution, and of PBS solution alone (yellow line) deposited on Ag dendrites. (b) SERS spectrum of Par j 4 (green line) on the CaF_2 substrate in the same spectral region. The different OH stretching modes are highlighted by green dashed lines.

In Figure S4, the CH stretching region of the allergen proteins, ranging from 2700 to 3100 cm^{-1}, is clearly visible in all the spectra. Furthermore, between 3150 and 3800 cm^{-1}, a complex pattern of O-H vibrational bands is observed, originating from both interfacial water molecules and the O-H and N-H groups of the proteins. In particular, Figure S4a shows a rearrangement in the hydrogen bond network typical of liquid water, characterized by three distinct bands with intensity ratios characteristic of interfacial water and confined water. These include the very low intense "network water" band around 3210 cm^{-1}, reflecting the highest degree of H-bond connectivity, the "intermediate water" band near 3490 cm^{-1}, which represents a distorted network commonly associated with a hydration shell,

and the "multimer water" band around 3562 cm⁻¹, corresponding to dimers or trimers (see references 21, 38 of the main text for details). A similar pattern is also observed in the spectrum of PBS, indicating that the phosphate buffered saline solution does not dry and remains in an aqueous environment confined within nanocavities. It is worth noting that, in the spectra of protein solutions, the hydration shell bands overlap with the O-H and N-H vibrations of the proteins, primarily at approximately 3345 cm^{-1}, as shown in Figure S4b, where the spectrum of Par j 4 in PBS, concentrated and left to dry onto CaF_2, and thus without the hydration shell, is provided as an example.

Figure S5: Raw SERS spectra

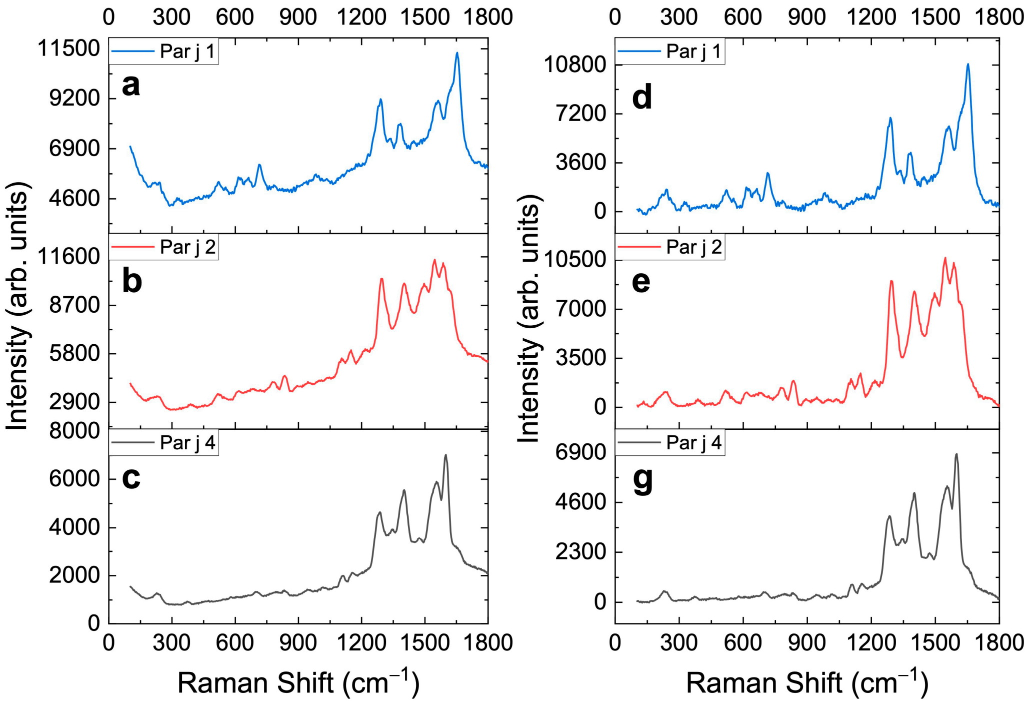

Figure S5. In panels (a, b, and c), the raw SERS spectra (without fluorescence background removal) of Par j 1 (blue line), Par j 2 (red line), and Par j 4 (black line) are shown, respectively, after drop-casting onto silver dendrites. In panels (d, e, and f), the corresponding SERS spectra, where the fluorescence background has been removed, are displayed as Par j 1, blue line, Par j 2, red line, Par j 4, black line. All the spectra are presented without undergoing any smoothing procedure.

Figure S5 displays the raw SERS spectra of allergenic proteins Par j 1, 2, and 4 before and after the fluorescence removal procedure. Notably, in these cases, the signal-to-noise ratio exceeds 40, as calculated using the formula provided in "Figure S2" section. The SERS peaks considered were those of the amide III band, while the background was measured between 3800 and 3900 cm^{-1}.

Figure S6: Reproducibility of SERS spectra

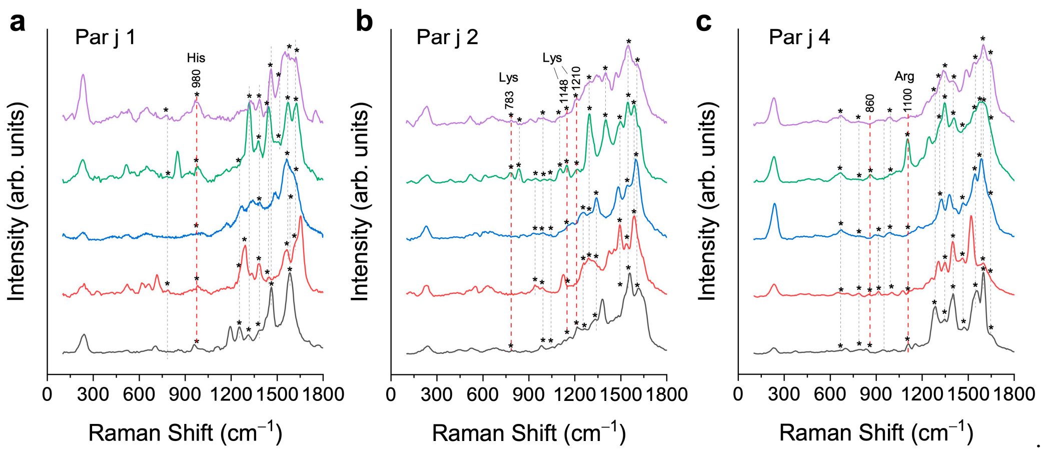

Figure S6. SERS spectra of Par j 1 (a), Par j 2 (b), and Par j 4 (c) in five different point of each sample in stacking view. The common signals are highlighted with "*" and the dashed lines.

Figure S6 presents a statistical analysis conducted on five points for Par j 1 (panel a), Par j 2 (panel b), and Par j 4 (panel c) proteins under identical experimental conditions. The vibrational modes of the most abundant amino acids are marked by red dashed lines. The results indicate that, for each allergen, the SERS vibrational frequencies remain consistent across all five spectra, while the intensity ratios can vary significantly. These variations arise from the different possible orientations of the molecules relative to the plasmonic substrate or their arrangement with respect to the nanocavity axes, where hot spot regions form. Along these axes, indeed, the molecular polarizability tensors experience the highest amplification effect. Furthermore, in the absence of functionalization, which would otherwise immobilize the molecules on the substrate, Par j proteins, enclosed within a hydration shell, remain free to rotate and move due to thermo-plasmonic effects and electrostatic interactions with the metal. Despite this variability, the observed spectral differences allow us to present a comprehensive and detailed overview of all the vibrational modes of the molecules. Furthermore, the trend of peak intensities relative to the amino acid abundance is preserved. By integrating the area of the lysine peaks in Par j 2 (around 780, 1148, and 1210 cm^{-1}) and comparing it to the same spectral ranges in Par j 1, across all five spectra recorded for each protein, we found that the intensities in Par j 2 were, on average, 5±1 times higher. This confirms that, despite the limited reproducibility of the spectra, the intensity trends remain consistent with the differing amino acid abundances.

Table S1: Amino acid abundance

Table S1. Abundance of each type of amino acid present in the primary structure of the Par j 1, 2, and 4 proteins.

Amino acid			Par j protein		
**Symbol**	**Name**	**Side chain**	**Par j 1**	**Par j 2**	**Par j 4**
G	Glycine	–H	13	8	8
A	Alanine	$-CH_3$	4	5	10

V	Valine	$–CH(–CH_3)_2$	9	7	2
P	Proline	$–CH_2–CH_2–CH_2–$	7	5	–
L	Leucine	$–CH_2–CH(–CH_3)_2$	7	4	4
I	Isoleucine	$–CH(–CH_3)–CH_2–CH_3$	4	8	8
C	Cysteine	$–CH_2–SH$	8	8	–
M	Methionine	$–CH_2–CH_2–S–CH_3$	4	2	4
F	Phenylalanine	$–CH_2–Ph$	1	3	5
W	Tryptophan	$–CH_2–(C_8H_6N)$	–	–	–
Y	Tyrosine	$–CH_2–Ph–OH$	2	2	–
S	Serine	$–CH_2–OH$	7	8	10
T	Threonine	$–CH(CH_3)–OH$	7	8	2
H	Histidine	$–CH_2–Im$	9	7	7
K	Lysine	$–CH_2–CH_2–CH_2–CH_2–NH_2$	11	15	6
R	Arginine	$–CH_2–CH_2–CH_2–NH–C(=NH)–NH_2$	3	4	6
D	Aspartic acid	$–CH_2–C(=O)–OH$	6	4	10
E	Glutamic acid	$–CH_2–CH_2–C(=O)–OH$	7	12	8
N	Asparagine	$–CH_2–C(=O)–NH_2$	2	1	2
Q	Glutamine	$–CH_2–CH_2–C(=O)–NH_2$	3	3	2
